# Nurse's Psychological Experiences of Caring for Severe COVID-19 Patients in Intensive Care Units: A Qualitative Meta-Synthesis

**DOI:** 10.3389/fpubh.2022.841770

**Published:** 2022-03-21

**Authors:** Peng Han, Xia Duan, Sijia Zhao, Xiaoping Zhu, Jinxia Jiang

**Affiliations:** ^1^Emergency Department, Shanghai Tenth People's Hospital, School of Medicine, Tongji University, Shanghai, China; ^2^Nursing Department, Shanghai First Maternity and Infant Hospital, School of Medicine, Tongji University, Shanghai, China; ^3^Nurisng Department, Shanghai Tenth People's Hospital, School of Medicine, Tongji University, Shanghai, China

**Keywords:** nurses, severe COVID-19 patients, intensive care unit, psychological experiences, meta-synthesis, qualitative systematic review

## Abstract

**Background:**

COVID-19 has been listed as an international public health emergency. During the pandemic, the nurses were affected physically and mentally when in contact with and caring for patients infected with COVID-19, especially those in intensive care units (ICUs).

**Objective:**

To summarize and evaluate the actual psychological experience of nurses caring for patients with severe pneumonia in the ICUs during the COVID-19 pandemic.

**Methods:**

Relevant publications were identified by systematic searches across 11 databases in December 2021. All qualitative and mixed-method studies in English and Chinese from 2019 that explored the experiences of nurses who cared for severe COVID-19 patients in ICUs were included. The qualitative meta-synthesis followed the Preferred Reporting Items for Systematic Reviews and Meta-Analyses (PRISMA) recommendations. Two independent reviewers selected the studies and assessed the quality of each study. Meta-synthesis was performed to integrate the results.

**Results:**

A total of 12 studies revealed 9 sub-themes and 3 descriptive themes: physical reactions and psychological changes, the need for support from multiple sources, and increased adaptation and resilience.

**Conclusion:**

Nurses who treated severe COVID-19 patients have experienced severe work trials and emotional reactions during the pandemic. They have also developed personally in this process. Managers should develop strategies that address the nurse's needs for external support, reasonably respond to public health emergencies, and improve nursing care outcomes.

## Introduction

The World Health Organization (WHO) declared the outbreak of the severe acute respiratory syndrome coronavirus 2 (SARS-CoV-2) that caused the 2019 coronavirus disease (COVID-19) as the public health emergency of international concern and characterized it as a pandemic (1). The virus is mainly transmitted through saliva droplets or discharged from the nose when an infected person coughs or sneezes or *via* the air through aerosols (2). The infection symptomatology varies drastically from no symptoms to life-threatening complications, including acute respiratory distress syndrome, multisystem organ failure, and death (3). Patients in critical condition have a greater risk of death and require intensive care (4). Treating and caring for critically ill patients is a difficult task with a high risk of infection.

The intensive care unit (ICU) was the primary venue for the treatment and nursing patients with severe COVID-19, which could provide advanced medical technology and special monitoring. An average of 25% (5–32% dependent on the institution and the country) of hospitalized patients were treated in the ICU (5, 6). In the early months of 2020, more than 42,000 medical staff supported Hubei province from other regions in China, nurses accounted for >60% of all medical staff (7). The nurses were the primary caregivers for COVID-19 patients in the ICU. They monitored the vital signs, collected specimens, provided nutritional support, carried out disinfection and other basic work, and also provided professional nursing such as non-invasive and invasive ventilation, conventional acute respiratory distress syndrome procedures, mechanical circulation support (ECMO), and coped with the disease changes occurring in patients at any time; hence, they are always considered a highly stressed group (5, 8, 9). Frontline nurses were directly exposed to the COVID-19 virus and were at high risk of infection without adequate protection (10). Moreover, faced with the high intensity of work, many of them worked long shifts for weeks without a sufficient number of days off, and their physical and mental health was at a disadvantage (11).

An increase in the amount and intensity of work was inevitable for nurses during the pandemic. In addition, they had to get accustomed to risks, practices, and new protocols (12). The WHO pointed out that healthcare professionals faced multiple psychosocial hazards during the COVID-19 pandemic, which could lead to fatigue, occupational burnout, increased psychological distress, and decreased mental health. These affected the health of the healthcare workers and the quality and safety of the care delivered (13). In addition, in similar crises, such as severe acute respiratory syndrome (SARS) and Middle East respiratory syndrome (MERS), nurses were exposed to severe stresses sources, including the fear of infection, stigma, and lack of human workforce and trust (14, 15). Some studies have pointed to higher rates of anxiety, depression, and posttraumatic stress disorder (PTSD) among nurses during and after the pandemics compared to other health care professionals (16, 17). This situation required close attention to the physical, psychological, and social requirements of nurses working under extremely stressful conditions, ensuring the advancement of nursing work (18, 19).

The emotions and stress experienced by nurses caring for severe COVID-19 patients may be related to their experience. The health departments of various countries and regions paid attention to the protection of nurses but were limited (10, 20). Thus, understanding nurse's experiences while treating patients in ICUs during the pandemic would help to understand their needs. The present study aimed to synthesize the research literature on the psychosocial experience of nurses caring for severe COVID-19 patients in the ICUs and point them in the direction of obtaining a comprehensive and effective support system during public health emergencies.

## Methods

### Design

This study aimed to identify, appraise, and synthesize data from qualitative studies that describe the psychosocial experience of caring for patients with severe COVID-19 from clinical nurse's perspectives. The Preferred Reporting Items for Systematic Reviews and Meta-Analysis (PRISMA) (21) was used as a basis for reporting the review. A meta-synthesis approach was used to combine and present the qualitative findings (22). Relevant articles were searched, and data were extracted and critically evaluated using a thematic synthesis based on the three steps outlined by Thomas and Harden (23): text coding line by line, developing descriptive themes, and generating analytical themes.

### Search Methods

Qualitative studies published from January 2019 to December 2021 in PubMed, Cochrane Library, CINAHL, Web of Science, Embase, Ovid, Elsevier, and Chinese databases, including Chinese National Knowledge Infrastructure (CNKI), Wanfang Database (CECDB), VIP Database, and China Biomedical Database (CBM), were searched by two authors in December 2021.

The search terms were developed, and subject headings were used where possible and adjusted for different databases. Four groups of keywords or MeSH terms were included and combined using Boolean operators: (1) nurs^*^; (2) COVID-19^*^, coronavirus disease 2019^*^, 2019-nCoV, coronavirus, covid pandemic; (3) severe case, serious illness, critical, intensive care unit, ICU, severe pneumonia (4) qualitative study^*^, qualitative research^*^, qualitative method^*^. To determine the eligibility of the potentially relevant studies, all titles and abstracts were reviewed by a researcher.

### Inclusion and Exclusion Criteria

#### Study Design(S)

The qualitative research or mixed-method studies from which qualitative data could be extracted, the primary qualitative research studies were included but were not limited to methodologies, such as phenomenology, grounded theory, action research, ethnography, and feminist research.

#### Participant(P)

Nurses that have taken care of severe COVID-19 patients in ICUs during the pandemic.

#### Interest of Phenomena(I)

Nurse's actual psychological experience of caring for patients with severe COVID-19. The psychological experience in this study was defined as the subjective experiences, perspectives, feelings, and views of the influences on mood status, cognitive-behavioral responses, and social factors of a person (24).

#### Context (Co)

Nurses had completed or were continuing to care for patients with severe COVID-19 in the ICUs.

#### Exclusion Criteria

Not qualitative research or collected qualitative data but analyzed using quantitative methods; Not written in English or Chinese; Not published in peer-reviewed journals, Case reports, conference proceedings, poster abstracts, and theses. Systematic reviews and other reviews were excluded, but their references were examined to identify a possible relevant study.

### Search Outcomes

**Figure 1:**
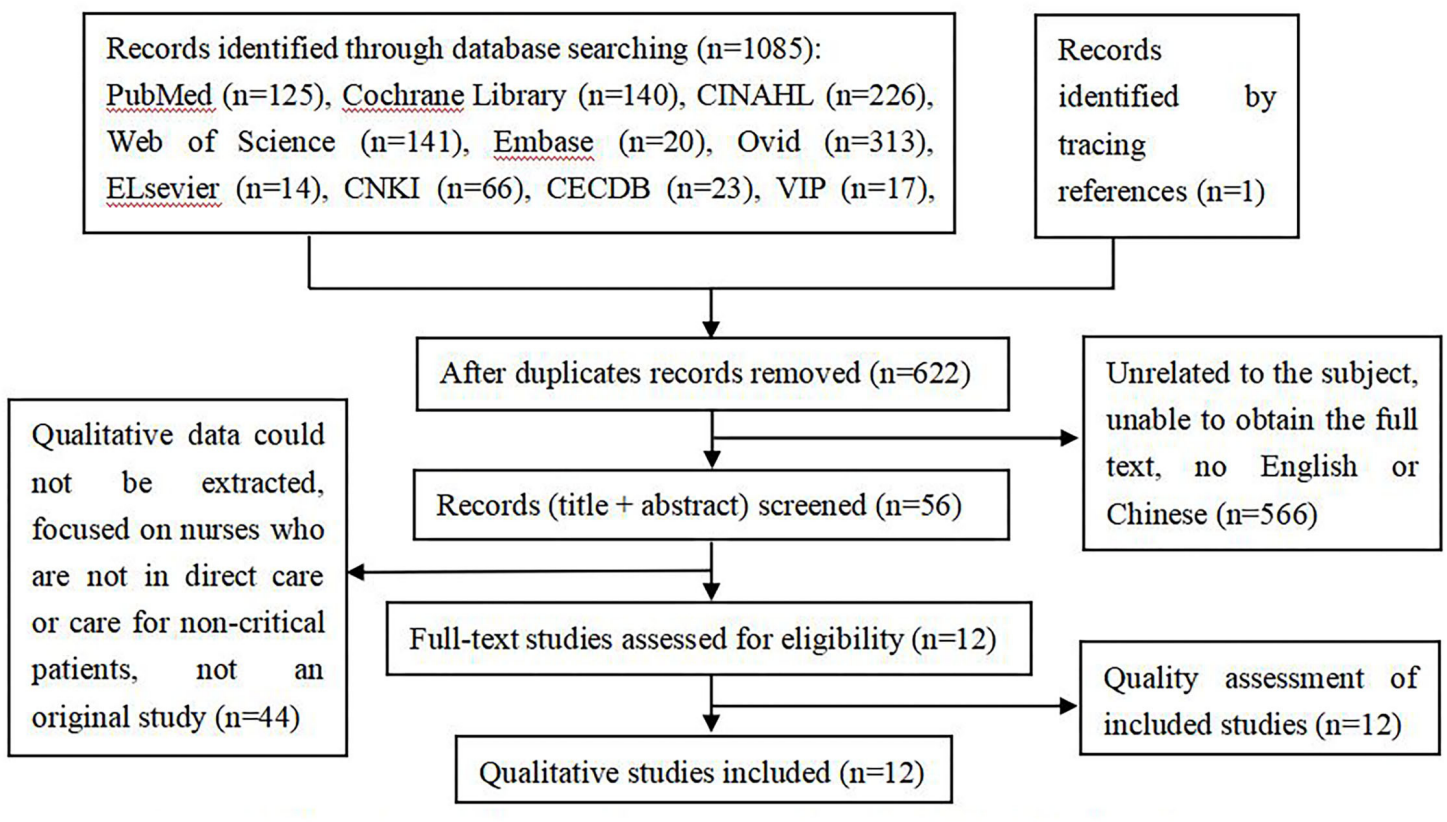
Flowchart of the search strategy and results (PRISMA flow diagram).

According to the inclusion and exclusion criteria, two researchers independently screened and extracted the literature. An initial search using the above strategy yielded a total of 1,085 articles. First, the title and abstract of the articles were read to exclude those unrelated to the subject, were repetitive, and full text could not be obtained. Subsequently, 566 articles were excluded. After reading the full text, 44 articles were excluded, and finally, 12 articles were identified as relevant, and one was traced from a reference. This search process is illustrated in Figure 1.

### Quality Appraisal

Two authors independently assessed the methodological quality of the 12 included studies. Initially, the authors worked independently using the Joanna Briggs Critical Assessment Tool for Methodological Quality Assessment (25). It consists of 10 questions designed to evaluate the studies quickly and efficiently with a simple yes, no, or unclear to each question. Each criterion was allocated a score (Yes = 2, No = 0, Unclear = 1), giving a total score of 20 for each study. These scores were then converted to a percentage. Subsequently, the results were discussed to reach a consensus, as all studies scored at least 70%, and none were excluded from the quality appraisal process (Table 1).

**Table 1:** Quality assessment of included studies in accordance with the criteria of the Joanna Briggs Critical Appraisal tool for qualitative research.

**Reference**	**Q1**	**Q2**	**Q3**	**Q4**	**Q5**	**Q6**	**Q7**	**Q8**	**Q9**	**Q10**	**Result (%)**
Tu et al. (26)	Y	Y	Y	Y	Y	N	Y	Y	Y	Y	18/20(90%)
Liu et al. (27)	Y	Y	Y	Y	Y	N	U	Y	U	Y	16/20(80%)
Shi et al. (28)	Y	Y	Y	Y	Y	N	Y	Y	U	Y	17/20(85%)
Guo et al. (29)	Y	Y	Y	Y	Y	Y	U	Y	U	Y	18/20(90%)
Muz et al. (12)	Y	Y	Y	Y	Y	N	N	Y	Y	Y	16/20(80%)
Su et al. (30)	Y	Y	Y	Y	Y	N	U	Y	Y	Y	17/20(85%)
Jiang et al. (31)	Y	Y	Y	Y	Y	N	U	Y	Y	Y	17/20(85%)
Gordon et al. (32)	Y	Y	Y	Y	Y	N	U	Y	Y	Y	17/20(85%)
Moradi et al. (33)	Y	Y	Y	Y	Y	N	U	Y	Y	Y	17/20(85%)
Chegini et al. (34)	Y	Y	Y	Y	Y	N	U	Y	Y	Y	17/20(85%)
Ozdemir et al. (35)	Y	Y	Y	Y	Y	N	U	Y	Y	Y	17/20(85%)
Fernandez-Castillo et al. (36)	Y	Y	Y	Y	Y	N	N	Y	Y	Y	16/20(80%)

*Critical appraisal (n = 10) of (Y, yes; N, no; U, unclear; NA, not applicable). Question, Q. Q1, Is there congruity between the stated philosophical perspective and the research methodology? Q2, Is there congruity between the research methodology and the research question or objectives? Q3, Is there congruity between the research methodology and the methods used to collect data? Q4, Is there congruity between the research methodology and the representation and analysis of data? Q5, Is there congruity between the research methodology and the interpretation of results? Q6, Is there a statement locating the researcher culturally or theoretically? Q7, Is the influence of the researcher on the research and vice-versa addressed? Q8, Are participants and their voices adequately represented? Q9, Is the research ethics according to the current criteria or for recent studies, and is there evidence of ethics approval by an appropriate body? Q10, Are the conclusions drawn in the research report arise from the analysis or interpretation of the data?*

### Data Extraction

A comprehensive study was conducted to characterize the quality of the content and assess the methodological development in the collected studies (37, 38). The extracted data included the author, the year of publication, country or region, research method, research subjects, interesting phenomena, and main research results. The results were cross-reviewed by two investigators, and any disagreement was resolved by discussion with a third investigator. These results are summarized in Table 2.

**Table 2:** Description of the included studies.

**References/** **Country**	**Research method**	**Participants**	**Aim**	**Results**
Tu et al. (26)/China	Descriptive qualitative research; semi-structured interviews	15 ICU nurses who had treated severe COVID-19 patients in Zhejiang province in China	To explore the true care experience of ICU nurses who have close contact with severe COVID-19 patients	8 themes in 3 stages: before participating in treatment: fear of inadequate self-protection, anxiety is not up to the task, a sense of vocation; participating in the treatment: nervousness and restlessness, quickly adapt to the intensive isolation ward into the treatment state, perception of lack of business knowledge; after participating in treatment: the symptoms of body discomfort were enlarged, stimulation of a sense of professional worth
Liu et al. (27)/China	Descriptive qualitative research; semi-structured interviews	12 ICU nurses who participated in the treatment of COVID-19 patients from a hospital in Beijing	To investigate the psychological status of ICU nurses at different stages during the treatment of COVID-19 in Hubei	8 themes in 3 stages: from receiving tasks to arriving in Wuhan: excitement and nervousness, lack of confidence; from arriving at the mission area to 4 weeks before work: fear and anxiety, frustration and helplessness, efforts to adapt to the situation; after the fifth week of the mission: missing family and tired, calm and confident, moved and grateful
Shi et al. (28)/China	Phenomenological approach; semi-structured diary analysis	9 nurses from a hospital in Jiangsu province in China who rushed to Wuhan's ICU ward in February 2020	To understand the changes of resilience of nurses who rushed to Wuhan's ICU under the COVID-19 epidemic, and to provide theoretical basis for nurses' psychological adjustment and intervention in public health emergencies	3 first-level themes and 8 second-level themes: Stress period (intrusive thoughts, physical challenges, psychological distress); Buffer zone (mobilization of psychological capital, stimulation of team resilience, understanding of social support); Reorganization (balance recovery, self-transcendence)
Guo et al. (29)/China	Phenomenological approach; semi-structured interviews	10 nurses worked in the isolation wards for severe COVID-19 patients in a hospital in Wuhan	To learn about the work experience of nurses in isolation wards for severe COVID-19 patients	Seven themes: sense of responsibility and mission, sense of achievement, feel the warmth of support, stress from work environment, stress of being infected, extreme physical exhaustion, loneliness and concern for family
Muz et al. (12)/Turkey	Phenomenological approach; semi-structured interviews	19 nurses who took care of COVID-19 patients in pandemic wards and pandemic intensive care units in tertiary public hospitals in Turkey	To reveal the experiences of nurses who care for COVID-19 patients during this process	Five themes: first meeting and getting caught unprepared, social isolation and loneliness, dilemma and conflict in professional roles, nursing: power born from difficulties and organizational expectations
Su et al. (30)/China	Phenomenological approach; semi-structured interviews	14 first-line nurses from a hospital in Beijing who had been dispatched to Wuhan, Hubei province, to fight COVID-19	To learn more about the true experience of first-line nurses caring for critically ill patients in remote emergency response to COVID-19	Four themes: heavy physical and mental burden, difficult observation of illness, psychological fluctuations, growth and harvest
Jiang et al. (31)/China	Phenomenological approach; semi-structured interviews	12 first-line nurses who had participated in the rescue of severe COVID-19 patients in Shanghai, China	To explore the experiences of nurses supporting the care of severe COVID-19 patient, to provide information and basis for nursing emergency rescue of public health emergencies	Four themes: strong sense of professional honor, heavy pressure, professional technology as support, support from all parties as motivation
Gordon et al. (32)/USA	Descriptive qualitative research; semi-structured interviews	11 ICU nurses who had cared for COVID-19 patients in the United States	To explore the experiences of critical care nurses working in central Texas amidst the pandemic	Five themes: emotions experienced, physical symptoms, care environment challenges, social effects, and short term coping strategies
Moradi et al. (33)/Iran	Descriptive qualitative research; semi-structured interviews	17 nurses worked in medical ICUs of a coronavirus (COVID-19) centre, Urmia, Iran	To explore the challenges experienced by ICU nurses throughout the provision of care for COVID-19 patients	Four themes: organization's inefficiency in supporting nurses, physical exhaustion, living with uncertainty and psychological burden of the disease
Chegini et al. (34)/Iran	Phenomenological approach; semi-structured interviews	15 nurses who provided care for patients infected by COVID-19 in critical care units of Iran's public hospitals	To describe the experiences of critical care nurses caring for patients infected by coronavirus disease 2019 (COVID-19)	Four themes: psychological challenges; organizational challenges; social challenges; professional challenges
Ozdemir et al. (35)/Turkey	Phenomenological approach; semi-structured interviews	10 cardiovascular nurses who were assigned to COVID-19 intensive care unit during the pandemic in Turkey	To explore the experiences of cardiovascular nurses working in a COVID-19 intensive care unit during the pandemic	Six themes: the duties and responsibilities in a COVID-19 intensive care unit; the differences of COVID-19 intensive care unit practices from cardiovascular practices; the transferrable skills of cardiovascular nurses in a COVID-19 intensive care unit; the difficulties encountered working in a COVID-19 intensive care unit; the difficulty of working with personal protective equipment; and the psychosocial effects of working in a COVID-19 intensive care unit
Fernandez-Castillo et al. (36)/Spain	Descriptive qualitative research; semi-structured interviews	17 ICU nurses from a tertiary teaching hospital in Spain	To explore and describe the experiences and perceptions of nurses working in an ICU during the COVID-19 global pandemic	Four themes: providing nursing care, psychosocial aspects and emotional lability, resources management and safety, professional relationships and fellowship

### Data Analysis and Synthesis

We used meta-aggregation to synthesize the findings of the qualitative studies. This is a method of systematic review that involves categorizing and re-categorizing of the synthesized findings of two or more studies (25).

First, each identified article was read multiple times to increase the familiarity and obtain a thorough understanding of the study aims, methods, and outcomes. Then, each discovery was extracted with the text data explaining or supporting the finding. The consistency between the research results and supporting data was evaluated by two researchers independently. Each finding provided some credibility: unequivocal, credible, or unsupported (25). The researchers studied the coded text to find the similarities and contradictions between these findings and descriptive data, each step was discussed by researchers to reach an intercoder agreement, and then created a classification to determine the meaning of the initial data set. For each theme, when needed, sub-themes were also developed following the same process. Finally, these categories were assessed repeatedly to identify the similarities and obtain synthesized results. In addition, emerged themes and sub-themes were evaluated in their occurrence by calculating the intra-study intensity and the inter-study frequency effect size to avoid under or overweighed themes and/or sub-themes.

## Results

The studies were conducted in the following countries: China (*n* = 6), Iran (*n* = 2), Turkey (*n* = 2), USA (*n* = 1), and Spain (*n* = 1). These 12 studies involved 161 nurses. All the included studies were descriptive qualitative analyses (*n* = 5) or phenomenological approaches (*n* = 7), wherein the data were collected by interviews. All studies published in 2020 or 2021 were original articles (Table 2). Three major themes emerged from the selected studies, reflecting the experience of nurses in caring for severe COVID-19 patients: physical reactions and psychological changes, the need for support from multiple sources, and increased adaptability and resilience. The themes were divided into several sub-themes of meaningful units, as demonstrated in Table 3.

**Table 3:** Thematic synthesis findings.

**Descriptive themes:**	**Sub-themes:**
Physical reactions and psychological changes	Physical symptoms caused by work characteristics
	Life-threatening pandemic induced anxiety
	Pressure to get into work
	Emotional reactions related to family
The need for support from multiple sources	Support and attention from the organization
	Longing for support outside of work
Increased adaptation and resilience	Gradual adaptation toward work
	Build trust with the patient
	Inspired professional values

### Theme 1: Physical Reactions and Psychological Changes

#### Physical Symptoms Caused by Work Characteristics

Overall, this review found that almost all studies reported various physical conditions among participants (12, 26, 28–36), including, but not limited to, sleep disturbances, headaches, damaged skin, exhaustion, and breathlessness. These conditions could be attributed to long working hours and high working intensity, and the treatment of critically ill patients increases the physical consumption of nurses. “*For patients requiring mechanical ventilation, strictly, we help them turn over and backslap every two hours, and have little time for rest at work”* (31); “*We are truly tired. In this ward, all female nurses are covered in spots because of stress, and some have hormonal disorders”* (33). Owing to the specificity of the infectious diseases, the protective equipment brought heavy burden and trouble to the nurses. “*Once, after putting on protective clothing, I had difficulty breathing, sweating, and felt unsteady to collapse”* (30); “*Due to the lack of protective equipment, we often do not eat or defecate in a shift, the whole body is wet. Protective masks also pressure forehead and face with a magic spell, too tired every day”* (29).

#### Life-Threatening Pandemic Induced Anxiety

Nurses were exposed to the virus in their workplaces, rendering them at high risk of infection. The nurses felt fearful and anxious. “*How can I not be nervous and worried? What if I get infected by spatter from a patient?”* (26); “*I'm worried about getting the disease, I'm worried about spreading it”* (32). As a result of negative news reports and other reasons, the grim situation and unknowns about the disease made nurses hypochondriac, worried that protection was not sufficiently safe. “*The stress caused by this disease has made me a little more aggressive, as I sometimes even become hostile toward my family, especially my brother”* (33); “*The number of bowel movements has increased in the last few days. The first symptom of novel coronavirus patients is diarrhea. Am I infected?”* (26).

#### Pressure to Get Into Work

While working in an isolated ICU was difficult, the repressive work environment made the nurses uncomfortable. “*The working environment is closed, and doctors are seldom in the ward. We have to communicate with the outside world through the pager. Sometimes in the face of emergency, nurses need to make independent decisions, which brings me great pressure”* (29). Specific protective equipment, such as screen filters and face masks, are required in the ICU, which could cause pressure injuries in nurses and be troubling. “*Deep indentations on my face after wearing the goggles for a day, I developed a pressure injury... My face suffered from severe eczema due to protective equipment, which was very itchy and uncomfortable. Fortunately, I had prepared medicine with me”* (31). The condition of severe patients changed quickly and the course of the disease was uncertain, which gave nurses great psychological pressure. “*At the beginning, patients often asked me about my illness, and I didn't know how to answer. For previous patients, I was very confident to tell him”* (30). Some of the nurses stated that they hesitated while providing care to the patients because of the fear of contamination and felt guilty because they believed that they were unable to provide adequate care. “*I was suffering from extreme remorse for shortening the duration of the patient's care. We were experiencing fear for ourselves even while taking the patient's meal to his room as his nutrition was dependent on us”* (12). Faced with deterioration or even death of severe COVID-19 patients, nurses felt overwhelmed and helpless, especially those who had little experience of death. “*If the patient is critically ill and cannot contact his family members, there is little hope for rescue, which will increase her discomfort. However, if do not do something, I will feel uncomfortable, and that feeling is especially helpless (Sigh)”* (30).

#### Emotional Reactions Related to Family

Similar to the medical team that helped Hubei in early 2020 in China, some nurses would be separated from their families for long periods. The prevalence of the disease and providing care for COVID-19 patients meant the loss of peace in life, and not being able to care for families made them worry about their familie's safety. “*The fear is that they will get infected, after all, other parts of Hubei are also seriously affected”* (29). Some nurses felt guilty and blamed themselves for the lack of care for their families. “*I have a 3-and-a-half-year-old son, I was feeling guilty when kissing him. In a way, I was blamed as a mom when I kissed my child, that affected me very bad”* (35). On the other hand, the long isolation made the nurses feel lonely and miss their families. “*It was fine when I first came here, but now I miss my children and parents when I have video calls with them (eyes red)”* (27).

### Theme 2: The Need for Support From Multiple Sources

#### Support and Attention From the Organization

Some nurses reported dissatisfaction with organizational support with respect to inadequate employee rights, poor planning, and a shortage of staff and protective equipment. “*There was no mask in the early days of the disease. We saw that disinfectant solutions were not in the ward and could not be found. The supply of gloves was reduced. Equipment was scarce”* (34); “*As a nurse, you are in an important place. You are always in contact with the patient, but you are always in the background in the system. I want my retirement rights and social rights”* (12); “*Since the outbreak of Coronavirus, no university deputies or hospital managers have come to ask “What are you doing here? What kinds of problems are you facing?” This shows that the system is not much concerned about personnel”* (33). However, many nurses also expressed that they received good organizational support that was helpful to their work. The common goal made the team cohesive, emphasizing the importance of organizational support. “*With professional training every two days, we are more confident in winning the battle against the epidemic”* (27). “*Your co-workers, they're along with you during this same crazy time… they are a huge support”* (32).

#### Longing for Support Outside of Work

Nurses believe that they need care and support from other people outside, for example, their families and friends, which could be a great spiritual boost during tough times. “*I was inspired by the fact that my family was proud of me”* (34). However, to the distress of some nurses, their families feared infection, and the lack of family support troubled the nurse. “*Our family are afraid that we might take the virus home and they could be infected. Their mentality is that we are all infected and could infect them all. It seems they fear us”* (33). Moreover, some nurses felt alienated and isolated from society because they were hospital workers. “*When we went to common areas in the hospital, there were complaints saying that we should not be there because we cared for COVID-positive patients. This type of social pressure wore us down a little”* (12). Strikingly, some nurses are stigmatized in life. “*You almost feel like the bubonic plague just walking around… that if someone touches you that they're gonna die instantly.”* (32). Some participants with sufficient social support thought building confidence is a great motivation. “*We are encouraged by the outpouring of support and donations from the public, we are not alone in fighting the epidemic”* (31). “*Over time, healthcare professionals were highly praised. In the first days, we were under a lot of pressure, but little by little, we were supported by the people and the government, and the healthcare professionals were introduced as heroes in the society, and this motivated us”* (34).

### Theme 3: Increased Adaptation and Resilience

#### Gradual Adaptation Toward Work

Facing COVID-19 for the first time was a big challenge for everyone. Many of the participants reported that over time, they adjusted to the work environment of the ICU and entered a treating state, their ability had been greatly improved (26–29, 35). “*I did have fear and anxiety in the early stage, but through psychological counseling and the help of my colleagues, I became calm and felt that I had changed from a medical nurse to an ICU specialist nurse”* (27). “*I have gained a lot here. I have not only learned a lot of professional knowledge, but also reflected on it. This is a process of positive motivation”* (30).

#### Build Trust With the Patient

The trust between the nurse and the patient was built during the nursing process, and the nurse's emotional temperature rose gradually. “*In the beginning, I also thought that I should contact with patients as little as possible. But gradually, I would no longer reject these patients and want to communicate with them more”* (30). The change in patient's attitudes toward nurses proved that they had done a good job. “*An old man did not cooperate with us at the beginning, but later he was moved by our behavior. From distrust at the beginning, to improvement later, he was very grateful to us”* (30).

#### Inspired Professional Values

The participants stated that the nursing profession had become stronger in this difficult period, and their motivation was strengthened when society understood the importance and meaning of nursing. “*During the pandemic, we proved to the society that the nursing profession is very important. At the moment, I think the society knows very well what we know, what training we have received, and our value”* (12). Participants and successfully treated critically ill patients by nurses, a heartfelt sense of responsibility, and mission were very critical, which could inspire professional values. “*I think it's a great honor for me to be selected by so many nurses, which is a full trust of the department, so I must fulfill my mission”* (30). Moreover, their professional maturity had increased, and the professional perception had changed through treatment work. “*We were the biggest part, namely nurses; while everyone shouldered responsibility, we put ourselves fully under that load. I realized that I was really a nurse”* (12).

## Discussion

A systematic review of 12 qualitative studies about the experiences of nurses who have treated of severe COVID-19 patients in ICUs, followed by a meta-synthesis, was performed on various databases after a manual search. The main findings indicated that the nurses face abundant physical and emotional stress while treating severe COVID-19 patients. These stressors arose from work burden, risk of infection, and public opinion. Nurse's coping strategies and external support improved their coping abilities under pressure, which improved the nursing work. Finally, through participation in the treatment of severe COVID-19 patients, nurses had improved their coping ability, including professional competence, communication skills, and the sense of professional value, which increased their levels of resilience and positive emotional experiences.

COVID-19 is easily spread through droplet transmission. For those with serious complications, the onset is rapid, causing surges in admissions that stretch the capacity of the health care systems, and if not properly addressed, endanger the patients and the hospital staff (39). According to a study in *The Lancet*, public health measures and supportive care (interventions developed and delivered largely by nurses) were the first and the only unequivocally effective defense against COVID-19 with no disease-specific prevention, treatment, or cure for COVID-19 (40). Therefore, nurses have earned well-deserved recognition for their essential roles in providing skilled, compassionate care for patients throughout this pandemic. ICUs are the main battleground for treating patients with severe COVID-19. The critical nursing team has a precise and skilled professional level in the treatment and care of critically ill patients and can grasp the operation skills of various rescue and life support equipment proficiently (41). During the pandemic, nurses gained positive and negative psychological experiences but always prioritized the patient's treatment. The professional quality of nurses, good cooperation of the team, and active guidance of nursing managers guaranteed the goal.

The results of the process of patient care in the ICUs showed that fear, worry, anxiety, depression, and other negative psychological experiences were common among nurses fighting COVID-19 in a critical situation, which affected their physical and mental health; the workload and intensity were the primary reasons. Similarly, at the beginning of the epidemic, several studies reported that nurses experienced emotions, such as fear and anxiety, because of the lack of up-to-date information on the causes of infectious diseases, their management, and ways of protection or continuously updating information (42, 43).

Since the nurses worked in a closed working environment, mental stress also arose from the pain of isolation. Measures, such as quarantine and social distancing, were applied to control the pandemic and reduce mortality and morbidity levels, causing social isolation and stigmatization (44). Social distancing and quarantine increased the nurse's fears and negatively affected their professional performance and psychological health (45). This influence was not conducive to nurse's family and social relations, which posed them with the psychological burden of being away from their families, children, and spouses and changing their habits.

The physical and mental pressure on the nurses effectuated by protective equipment could not be ignored, and special equipment added to the burden. The results showed that they experienced physical symptoms, such as dyspnea, headache, muscle pain, and excessive sweating, because of the use of personal protective equipment, which consequently increased their stress. The accuracy of nursing operations was reduced and communication with patients or colleagues was obstructed, resulting in anxiety and frustration among nurses. In addition, wearing face screens, goggles, and masks for long periods caused pressure injury to their faces. However, to control contagious diseases, protective equipment and training of healthcare workers are critical for maintaining a safe working environment (46). These findings highlighted that providing adequate ergonomic protective equipment is essential.

Social support refers to the social resources provided by formal or informal support groups that individuals perceive subjectively and/or receive objectively (47). This review demonstrated that the nurses need support from multiple sources. Organizational support should be based on the interests of nurses. Importantly, nurses working in the event of an epidemic should be made to feel valuable. Their safety should be a priority, and they should be appropriately rewarded to provide positive support when a similar situation occurs in the future (48, 49). Notably, at the beginning of the pandemic, the uncertainty of assigning tasks and measures was exhausting for the nurses. This finding suggested that healthcare facilities, such as hospitals providing wards during disasters and emerging infectious diseases, need to plan for crisis management, including epidemic prevention, preparedness, and response processes (12). External support includes religious beliefs, friends, information from the environment, and support from colleagues, family, or social circle (47). When colleagues encounter difficulties, team cooperation and mutual support are critical to improving the state of mind. Family members and friends are often able to understand the nurse's situation, and their persuasion and comfort are focused and effective.

A systematic review revealed that insufficient social support was one of the risk factors for developing negative psychological consequences among healthcare professionals and providers during disasters (50). Outside the workplace, while mainstream media extolled nurses as heroes, some nurses endured stigmatizing attitudes by those viewing them as virus carriers. Therefore, community support for nurses is crucial during an epidemic (51), and policy-makers should address the barriers that create ethical challenges for nurses fighting COVID-19 (52). The study showed that a high level of social support and recognition for healthcare workers in public health emergencies could be shealing (53). In view of the external pressure, the relevant departments should actively guide the media, avoid the emergence of untrue reports, establish a good public image of medical staff, and consider outstanding medical staff as examples to promote positive energy.

Along with the negative psychological impact of COVID 19, positive emotions, such as confidence, inner satisfaction, professional pride, and commitment to the profession, are also reported in the results. Positive psychology mainly studies personality traits such as wisdom, courage, enthusiasm, and gratitude. Resilience means the ability to bounce back or recover easily when confronted by adversity, trauma, misfortune, or change (54). Thus, cultivating the positive strength of personality ensures that individuals acquire good resilience (55). Also, there is a need for self-actualization in everyone's heart, which stimulates people's positive power and excellent qualities. The key point of resilience is to adapt to various environments. Positive psychological strength and excellent psychological qualities improve adaptability (56). Therefore, positive psychological quality and resilience are interrelated. As described by Jnah and Robinson (57), the positive emotions and self-efficacy of nurses exert a positive effect on the improvement of their resilience, indicating a high degree of confidence in the face of difficulties. Hence, psychological interventions are essential to increase the mindfulness and resilience of the nurses and their families (58, 59).

Furthermore, the resilience of nurses is a positive psychological quality, which plays a critical role in response to public health emergencies. High resilience makes the nurses competent and increases their patriotism and reverence for life (60). While saving lives, they gain a sense of self-worth as well as professional benefits. Several effective strategies have been proposed to help nurses improve their organizational support, cope with negative emotions, and improve resilience. A multimodal resilience training program improves individual resilience and psychological outcomes, such as symptoms of anxiety, depression, burnout syndrome, and posttraumatic stress disorder (PTSD). The strategies include a two-day educational workshop, written exposure sessions, event-triggered counseling sessions, mindfulness-based stress reduction exercises, and a protocol-based aerobic exercise regimen (61). The Stress Management and Resiliency Training (SMART) program encompass attention training and practice of gratitude, empathy, higher meaning, and forgiveness (62). Moreover, music therapy and online mind-body skill training are effective in improving nurse's resilience (63). Nursing managers focus on the psychological status of nurses in order to establish organizational strategies.

## Conclusion

The findings of this review suggested that nurses working in critical care units during the COVID-19 pandemic experience psychological and physical distress as they cope with their work, social relationships, and personal lives. Thus, the active involvement of governments, policymakers, nursing groups, and healthcare organizations in supporting nurses during and after a pandemic or epidemic is essential to improve professional satisfaction and ensure the sustainability of the nursing workforce. Future studies will focus on the long-term psychological experience of nurses treating patients with severe COVID-19 and on strategies that can provide a better work experience. It is speculated that these results can act as a guide to understanding nurse's real feelings and needs that would contribute to further studies to be better prepared and improve the quality of nursing when responding to future public health emergencies.

## Limitations

This meta-synthesis has several limitations. According to the inclusion criteria, only primary qualitative studies published in indexed journals in English or Chinese were selected. Therefore, gray literature and dissertations were not searched, which might have introduced an information bias. The response to the pandemic in different countries may lead to various protocols and policies that might influence the nurse's attitudes and work experiences. Finally, this meta-synthesis represents the authors and other researchers with different interests, which might provide varied results.

## Data Availability Statement

The raw data supporting the conclusions of this article will be made available by the authors, without undue reservation.

## Author Contributions

PH: conceptualization, methodology, formal analysis, writing—original draft, and writing—review and editing. XD: conceptualization, methodology, writing—original draft, and writing—review and editing. SZ: conceptualization, methodology, formal analysis, and writing—review and editing. XZ and JJ: methodology, formal analysis, and writing—review and editing. All authors contributed to the article and approved the submitted version.

## Funding

This study was supported by Shanghai Shenkang Hospital Development Center Clinical Science and Technology Innovation Project (SHDC12021611).

## Conflict of Interest

The authors declare that the research was conducted in the absence of any commercial or financial relationships that could be construed as a potential conflict of interest.

## Publisher's Note

All claims expressed in this article are solely those of the authors and do not necessarily represent those of their affiliated organizations, or those of the publisher, the editors and the reviewers. Any product that may be evaluated in this article, or claim that may be made by its manufacturer, is not guaranteed or endorsed by the publisher.
